# Time-restricted feeding alters isoflurane-induced memory deficits

**DOI:** 10.1515/tnsci-2020-0130

**Published:** 2020-09-21

**Authors:** Jia Song, Shuaishuai Chu, Xin Fang, Fangxia Xu, Peng Zhang, Xin Zhao, Zhengliang Ma, Tianjiao Xia, Xiaoping Gu

**Affiliations:** Department of Anesthesiology, Affiliated Drum Tower Hospital of Medical Department of Nanjing University, 321 Zhong Shan Road, Nanjing, Jiangsu 210008, P. R. China; Department of Anesthesiology, Nanjing Stomatological Hospital, Medical School of Nanjing University, No 30 Zhongyang Road, Nanjing, 210008, China; Jiangsu Key Laboratory of Molecular Medicine, Nanjing University, Nanjing 210093, China

**Keywords:** aligned/misaligned feeding, daily rhythm, cognition dysfunction, isoflurane

## Abstract

Food consumption during the rest phase promotes circadian desynchrony, which is corrected with harmful physiological and mental disorders. Previously, we found that circadian desynchrony was involved in isoflurane-induced cognitive impairment. Here, we scheduled food access to modulate daily rhythm to examine its impact on isoflurane-induced cognitive impairments. Mice were randomly transferred to restricted feeding (RF) time groups: Control group (Zeitgeber time (ZT) 0–ZT24, *ad libitum* feeding), Day-Feeding group (ZT0–ZT12, misaligned feeding), and Night-Feeding group (ZT12–ZT24, aligned feeding). Then, some of them were subjected to 5 h of 1.3% isoflurane anaesthesia from ZT14 to ZT19 and were divided into the Control + Anes group, the Day-Feeding + Anes group, and the Night-Feeding + Anes group. Mini-Mitter was used to monitor the daily rhythm. Fear conditioning system was conducted to assess cognition of mice. We observed that the Night-Feeding group adapted to RF gradually, whereas the Day-Feeding group exhibited a disturbed daily rhythm. The Night-Feeding + Anes group exhibited a partially enhanced daily rhythm, whereas the Day-Feeding + Anes group exhibited sustained phase advances and diurnality score increase 7 days after isoflurane anaesthesia. Notably, in tests of hippocampus-dependent contextual memory, the Night-Feeding + Anes group demonstrated decreased deficits; the Day-Feeding + Anes group showed prolonged post-anaesthetic deficits 14 days after isoflurane anaesthesia. However, amygdala-dependent cued-fear conditioning post-anaesthesia was not altered by the RF schedule. In conclusion, we demonstrated that misaligned feeding disturbed the daily rhythm and led to persistent post-anaesthetic cognitive dysfunction. Aligned feeding enhanced the daily rhythm partially and improved post-anaesthetic cognitive dysfunction.

## 1 Introduction

Synchronizing internal rhythmicity with environmental cues is essential for the optimal adjustment of the body to predictable daily challenges. It has long been recognized that light is the primary zeitgeber for the central clock in the suprachiasmatic nucleus (SCN) of the brain [1]. Evidence is accumulating that the timing of food availability is an important entrainment signal for circadian clocks outside the SCN [2]. A restricted feeding (RF) schedule has been well studied in rodent models [3,4], in which the food availability time is restricted for several hours per day. Food consumption during the rest phase can deliver contradicting inputs to the circadian timing system [5].

Circadian rhythm disruption is emerging as a new risk factor for cognitive dysfunction in clinical and rodent investigations. Clinical investigations have found significant cognitive impairments in long-time shift workers [6]. Sprecher and Videnovic demonstrated that circadian oscillation is disturbed in neurodegenerative diseases and that chronobiological interventions can be used therapeutically [7,8]. Desynchronization or misalignment of circadian oscillators causes decrements in cognitive function, as observed in animal models. It was reported that experimental jet-lag conditions caused acute and persistent spatial memory deficits in hamsters [9]. The use of chronobiological manipulation of scheduled feedings as reinforcements of circadian rhythms has been shown to alleviate cognitive impairments in Alzheimer’s, Parkinson’s, and Huntington’s models [10,11,12].

In our previous study, we found that the daily rhythm was involved in isoflurane-induced cognitive impairment in mice [13]. Therefore, we hoped to find a manoeuvrable procedure without genetic, pharmacological, or surgical interventions to modulate the daily rhythm and thereby relieve memory decline. We hypothesized that misaligned/aligned feeding might play a significant role in post-anaesthetic cognitive modifications by modulating the daily rhythm.

## 2 Materials and methods

### 2.1 Experimental animals

Two-month-old male C57BL/6J mice (Model Animal Research Center of Nanjing University, China) were housed in the same room, with a constant temperature (22 ± 2°C) and humidity (55% ± 5%). The light intensity in the housing room was about 150–200 lux. There were six mice in one cage during the adaptation period. The mice were acclimatized to a 12 h:12 h light:dark (LD) cycle for 3 weeks before any interventions were undertaken. The objective time was defined as the zeitgeber time (ZT) rather than external time. Lights on at 8:00 was defined as ZT 0, and lights off at 20:00 was defined as ZT 12. Food was available *ad libitum* during the adaptation period and was scheduled in the experimental period. Water was available *ad libitum.*

### 2.2 Ethical agreement

All procedures were conducted in accordance with the applicable laws and guidelines regarding animal care and were approved by the Nanjing Drum Tower Hospital Experimental Animals Welfare and Ethical Committee (No. 20171102). All processing methods were conducted in accordance with Directive 2010/63/EU. This manuscript adheres to the applicable EQUATOR guidelines.

### 2.3 Scheduled food access

All mice were fed freely for 3 weeks and then transferred into different restricted time feeding groups. Mice in the Control group were given access to the food chamber from ZT 0 to 24; then some of them received isoflurane anaesthesia and were placed in the Control + Anes group randomly. Mice in the Night-Feeding group were given access to the food chamber during the dark phase from ZT12 to 24, which aligned with their active phase, and then some of them received isoflurane anaesthesia and were placed in the Night-Feeding + Anes group. Mice in the Day-Feeding group were given access to the food chamber during the light phase from ZT0 to 12, which misaligned with their active phase, and then some of them received isoflurane anaesthesia randomly and were placed in the Day-Feeding + Anes group.

### 2.4 Anaesthesia protocol

Mice were placed in plexiglass chambers, exposed to a mixture of 1.3% isoflurane (Lunan Better Pharmaceutical Co., Ltd, 64150704) in 100% oxygen at a flow rate of 2–3 L/min for 5 h from ZT14 to ZT19; thus, we manipulated the mice anaesthesia during the activity period. The concentrations of isoflurane were continuously monitored with an anaesthesia gas monitor.

### 2.5 Fear conditioning system (FCS)

We used a FCS (Panlab, Harvard Apparatus, Spain) to test hippocampus-dependent contextual memory and amygdala-dependent cue memory. Mice were trained and tested only once to avoid the effects of prior manipulation. To avoid daily rhythm in performance on this test, we conducted FCS at the same time of day (from ZT0 to ZT2) in all groups. Each mouse was allowed to explore the FCS chamber for 5 min before presentation of a 2 Hz pulsating tone (80 dB, 4,000 Hz), which persisted for 60 s. The tone was immediately followed by a mild foot shock (0.8 mA for 0.5 s). Twenty-four hours later, each mouse was allowed to stay in the same chamber for a total of 300 s. The tone test was performed 60 min after the end of the contextual test. Each mouse was allowed to stay in a different chamber for a total of 360 s. The same tone was presented for the second 180 s without the foot shock. Cognitive function during the test was assessed by measuring the amount of time the mouse demonstrated “freezing behaviour” (freezing time), which was defined as a completely immobile posture (except for respiratory efforts), using the PACKWIN software.

### 2.6 Measurements of daily rhythm

As previously described [13], a radio transmitter (G2 E-Mitter; Mini Mitter Co. Inc., A Respironics Company, Bend, OR, USA) was planted into the abdomen of each mouse to record the rest-activity and core body temperatures of the mice. Each mouse was housed in an individual cage on the receiver board (ER-4000 receiver). All receivers were connected to a computer with the VitalView Data Acquisition System (version 4.2; Respironics, Inc.). The data were recorded in 6 min intervals. After at least a 2 week recovery from surgery, the mice were then used in the experiments.

### 2.7 Experimental design

#### 2.7.1 Experiment 1

**Figure 1:**
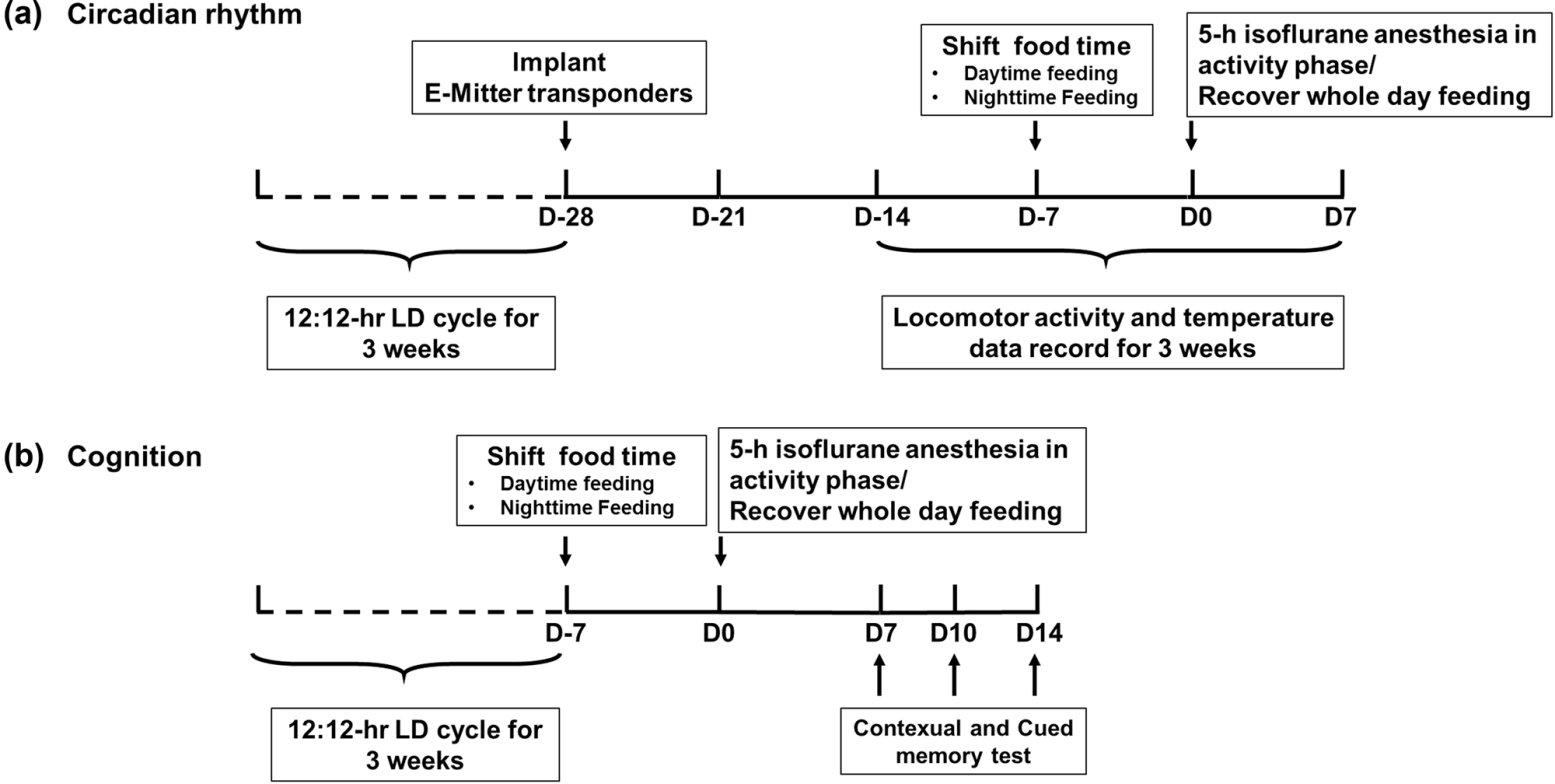
(a) The effect of feeding schedule on daily rhythm before and after isoflurane anaesthesia. After manipulating RF, mice were divided into five groups: control group (feeding throughout the entire day, from ZT0 to ZT24, *n* = 8), Night-Feeding group (feeding during nighttime, from ZT12 to ZT24, *n* = 8), Day-Feeding group (feeding during daytime, from ZT0 to ZT12, *n* = 8), Night-Feeding + Anes group (feeding during nighttime from ZT12 to ZT24 and receive isoflurane on D0, *n* = 8), and Day-Feeding + Anes group (feeding during daytime from ZT0 to ZT12 and receive isoflurane on D0, *n* = 8). (b) The effect of feeding schedule on post-anaesthetic fear memory at D7, D10, D14. After manipulating RF, mice were divided into six groups: Control group (feeding throughout the entire day, from ZT0 to ZT24, *n* = 8), Control + Anes group (feeding throughout the entire day, from ZT0 to ZT24 and receive isoflurane on D0, *n* = 8), Night-Feeding group (feeding during nighttime, from ZT12 to ZT24, *n* = 8), and Day-Feeding group (feeding during daytime, from ZT0 to ZT12, *n* = 8), Night-Feeding + Anes group (feeding during nighttime from ZT12 to ZT24 and receive isoflurane on D0, *n* = 8), Day-Feeding + Anes group (feeding during daytime from ZT0 to ZT12 and receive isoflurane on D0, *n* = 8).

To examine the effect of RF on the isoflurane anaesthesia-induced locomotor activity and core body temperature rhythms, 40 mice were randomly divided into five groups, and each group was treated, as described previously in Section 2.2 Scheduled food access (Figure 1a). All mice were put into cages to monitor the gross motor activity using a Mini-Mitter from the third week after the wireless telemetry was implanted. The data recording ended 1 week after isoflurane anaesthesia. To minimize the usage of mice, we did not use a Control group in this experiment; instead, we recorded 7 days before the RF as the Control baseline (D-pre).

#### 2.7.2 Experiment 2

To examine the effect of RF on isoflurane anaesthesia-induced memory deficits, 144 mice were divided randomly into six groups, and each group was treated, as described previously in Section 4.2 Scheduled food access (Figure 1b). We used an FCS to test the contextual and cued memory on D7, D10, and D14 after isoflurane anaesthesia.

### 2.8 Statistical analyses

All data were expressed as the mean ± SD (standard deviation). In the FCS test, one-way ANOVA, followed by Bonferroni’s multiple comparisons test, was applied at each time point. Daily rhythm analysis was performed by fitting a cosinor function to the data using the Chronos-Fit program for data analysis. This program fits a cosine curve to the measured data points and calculates the following statistics of rhythm: acrophase (peak time of the rhythm), amplitude (half of the peak-to-trough of the rhythmic change), MESOR (a rhythm-adjusted 24 h mean), and diurnality score (light/dark ratio). For clarity, amplitude and MESOR were calculated as the percentage from the baseline and phase shift for acrophase. For each row parameter examined, two-way ANOVAs (Feeding conditions × Anaesthesia) with repeated measures were conducted, followed by Bonferroni post hoc analysis. The statistical analysis was performed using the SPSS 20.0 software (IBM Corporation, Armonk, NY). The differences were considered statistically significant at the level of *P* < 0.05.

## 3 Results

### 3.1 RF and isoflurane anaesthesia did not change the weight of mice

We measured body weight from the first day after shifting the feeding protocol until 7 days after isoflurane anaesthesia (Table 1). The body weights did not significantly differ between the treatment groups throughout the duration of scheduled feedings and 7 days after anaesthesia.

**Table 1:** Body weight during the 7-day scheduled feeding period and 7 days after anaesthesia

	Control	Control + Anes	Day-Feeding	Day-Feeding + Anes	Night-Feeding	Night-Feeding + Anes
D-7	22.40 ± 0.51	22.90 ± 1.11	22.41 ± 0.82	22.51 ± 0.91	22.51 ± 1.42	22.78 ± 1.31
D-6	22.10 ± 1.42	22.91 ± 1.10	22.61 ± 1.20	22.82 ± 1.02	22.91 ± 1.12	22.69 ± 1.21
D-5	22.11 ± 1.21	22.92 ± 1.09	22.51 ± 1.22	22.71 ± 1.22	22.61 ± 1.11	22.79 ± 1.01
D-4	22.32 ± 1.11	22.92 ± 1.39	22.71 ± 1.23	22.82 ± 1.22	22.61 ± 0.81	22.89 ± 1.51
D-3	22.61 ± 1.31	23.12 ± 1.15	22.91 ± 1.12	22.82 ± 1.31	23.21 ± 0.72	22.79 ± 1.21
D-2	22.92 ± 1.20	22.89 ± 0.89	22.69 ± 1.29	23.42 ± 1.02	22.42 ± 0.91	23.81 ± 0.81
D-1	23.12 ± 1.29	22.91 ± 1.01	22.82 ± 1.28	23.43 ± 1.12	22.19 ± 0.81	23.32 ± 1.21
D1	23.09 ± 1.22	22.92 ± 1.02	22.12 ± 1.31	23.33 ± 1.21	22.22 ± 0.92	23.43 ± 1.01
D2	23.54 ± 1.31	23.23 ± 1.03	23.13 ± 1.32	23.63 ± 0.91	22.52 ± 0.92	23.32 ± 0.92
D3	23.52 ± 1.31	23.32 ± 0.89	23.21 ± 1.22	23.63 ± 0.92	22.69 ± 1.11	23.61 ± 0.89
D4	23.51 ± 1.31	23.71 ± 0.89	23.51 ± 1.21	23.63 ± 0.92	22.89 ± 1.23	23.09 ± 1.82
D5	23.71 ± 1.41	23.75 ± 0.81	23.52 ± 1.11	23.81 ± 0.91	23.09 ± 1.01	23.59 ± 0.89
D6	23.92 ± 1.51	23.72 ± 0.71	23.61 ± 1.21	23.62 ± 1.12	22.72 ± 1.18	23.79 ± 0.88
D7	24.09 ± 1.61	23.81 ± 0.41	23.71 ± 1.52	23.81 ± 1.13	23.41 ± 1.02	23.59 ± 0.92

### 3.2 Misaligned feeding disrupted pre-anaesthetic daily rhythm levels

#### 3.2.1 Misaligned feeding disrupted pre-anaesthetic rest-activity rhythm

**Figure 2:**
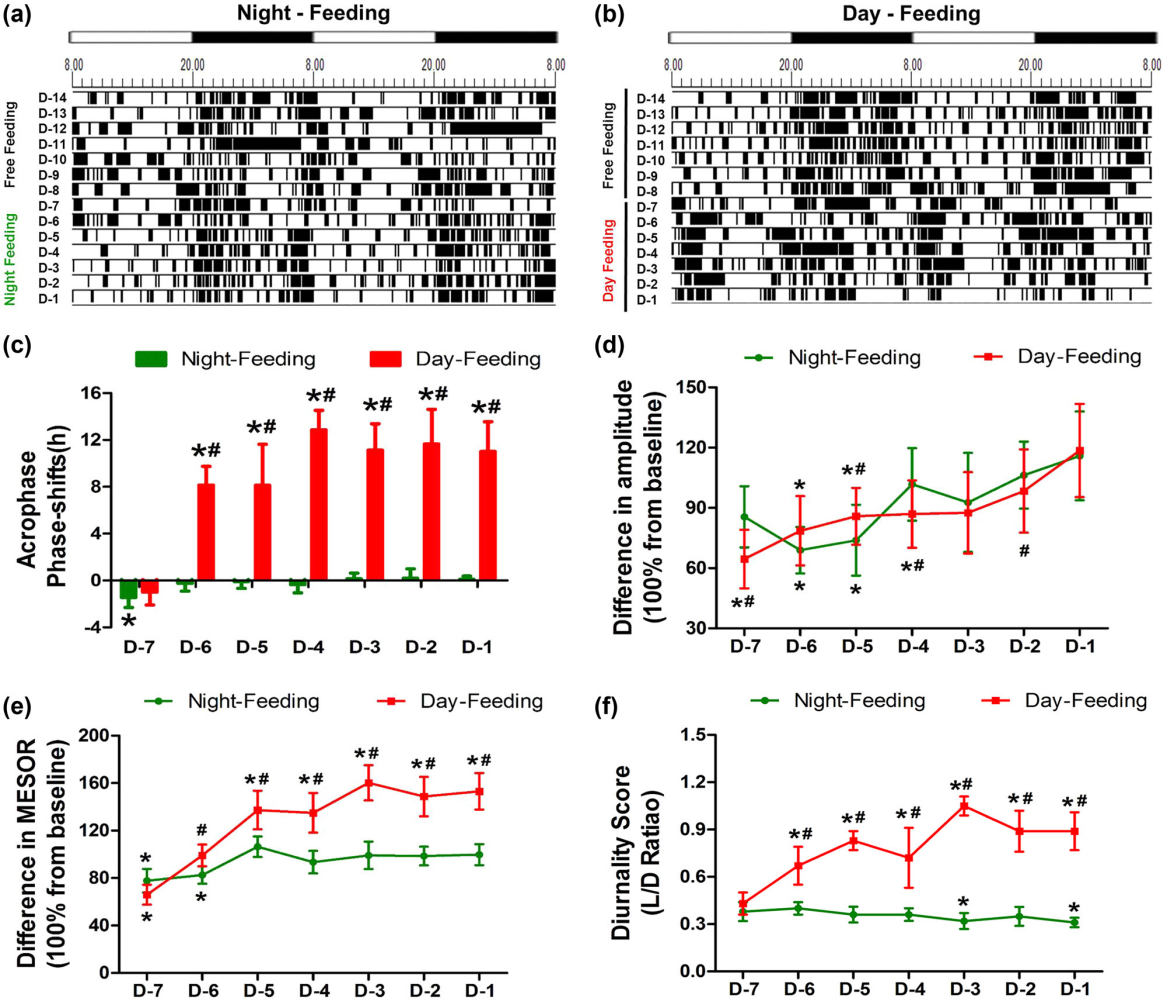
The effect of feeding schedule on rest-activity rhythm during 7 days between the Night-Feeding group and the Day-Feeding group. (a and b) Representative double-plotted actograms show the activity patterns after feeding schedule in a 12 h:12 h light:dark cycle. The black sections in the actograms represent a greater amount of activity than the average value in 48 h, while the white ones represent less activity, the abscissa 8.00 means ZT0; 20.00 means ZT12. (c) Phase shift of acrophase. (d) Amplitude. (e) MESOR. (f) Diurnality Score. *P < 0.05 vs mean values in 7-day pre-feeding period; ^#^ *P* < 0.05 vs Night-Feeding group. Positive values represent phase advance, *n* = 8.

The activity patterns of the mice were analysed with Chronos-Fit and displayed as double-plotted actograms (Figure 2a and b). The black sections in the actograms represent a greater amount of activity than the average value over 48 h, while the white sections represent less activity. The rhythm of locomotor activity exhibited different trends of change between the Day-Feeding and the Night-Feeding mice. After several days of fluctuation, the Night-Feeding group adapted to the shift in food access gradually. The Day-Feeding group showed increased activity durations during the daytime and decreased activity durations at night, suggesting that their nocturnality was disrupted. We used four parameters to represent the daily rhythm: acrophase, amplitude, MESOR, and diurnality score. The Night-Feeding group exhibited a phase delay on D-7 (18.29 ± 0.36, *P* < 0.001), which reversed on D-6. The Day-Feeding group demonstrated a phase advance to phase-inverted over time, compared with the group’s own baseline (D-6, 10.07 ± 1.46, *P* < 0.001; D-5, 10.08 ± 3.31, *P* < 0.001; D-4, 5.34 ± 1.53, *P* < 0.001; D-3, 7.07 ± 2.14, *P* < 0.001; D-2, 6.56 ± 2.78, *P* < 0.001; and D-1, 7.19 ± 2.43, *P* < 0.001) (Table 2). The Night-Feeding group demonstrated a decreased amplitude on D-6 (37.94 ± 4.06, *P* < 0.001) and D-5 (40.25 ± 4.93, *P* = 0.001), which reversed on D-4. The Day-Feeding group exhibited a decreased amplitude of the rest-activity rhythm on D-7–D-5 (D-7, 33.39 ± 3.34, *P* < 0.001; D-6, 40.97 ± 5.63, *P* = 0.005; D-5, 44.96 ± 5.04, *P* = 0.024), which then reversed on D-4. The Night-Feeding group exhibited a decreased MESOR of the rest-activity rhythm on D-7 (73.85 ± 6.19; *P* < 0.001) and D-6 (78.7 ± 6.27; *P* < 0.001), which then reversed to baseline on D-5. The Day-Feeding group demonstrated a significantly decreased MESOR of the rest-activity rhythm on D-7 (62.72 ± 4.84; *P* < 0.001), which increased on D-5–D-1 (D-5, 130.75 ± 10.44, *P* < 0.001; D-4, 128.54 ± 11.81, *P* < 0.001; D-3, 152.59 ± 4.85, *P* < 0.001; D-2, 141.41 ± 7.02, *P* < 0.001; and D-1, 145.69 ± 4.89, *P* < 0.001). The Night-Feeding group displayed a decreased diurnality score on D-3 and D-1. The diurnality score of the Day-Feeding group increased over the 7 days.

**Table 2:** Results of pre-anaesthetic cosinor analysis after RF

	Locomotor activity		Temperature	
	Night-Feeding	Day-Feeding	Night-Feeding	Day-Feeding
Acrophase				
D-Pre	18.29 ± 0.36	18.21 ± 0.53	18.36 ± 0.25	18.31 ± 0.54
D-7	19.72 ± 0.67	19.20 ± 0.84	20.69 ± 0.61	20.91 ± 0.81
D-6	18.53 ± 0.53	10.07 ± 1.46	20.23 ± 0.19	7.85 ± 1.27
D-5	18.38 ± 0.55	10.08 ± 3.31	18.49 ± 0.26	9.33 ± 1.26
D-4	18.64 ± 0.71	5.34 ± 1.53	18.43 ± 0.34	5.45 ± 1.62
D-3	18.15 ± 0.45	7.07 ± 2.14	18.33 ± 0.28	8.75 ± 1.80
D-2	18.11 ± 0.73	6.56 ± 2.78	18.31 ± 0.21	7.13 ± 1.37
D-1	18.19 ± 0.28	7.19 ± 2.43	18.16 ± 0.41	7.92 ± 1.39
Amplitude				
D-Pre	56.13 ± 8.68	53.28 ± 7.85	0.79 ± 0.08	0.79 ± 0.08
D-7	46.9 ± 4.30	33.39 ± 3.34	0.61 ± 0.08	0.58 ± 0.13
D-6	37.94 ± 4.06	40.97 ± 5.63	0.51 ± 0.08	0.63 ± 0.13
D-5	40.25 ± 4.93	44.96 ± 5.04	0.51 ± 0.08	0.61 ± 0.14
D-4	55.69 ± 2.63	45.38 ± 5.56	0.74 ± 0.09	0.77 ± 0.14
D-3	50.24 ± 5.96	45.62 ± 7.85	0.71 ± 0.10	0.77 ± 0.15
D-2	58.65 ± 6.51	51.33 ± 7.08	0.71 ± 0.09	0.70 ± 0.14
D-1	63.31 ± 3.55	61.56 ± 5.00	0.71 ± 0.08	0.71 ± 0.14
MESOR				
D-Pre	95.57 ± 5.92	95.79 ± 7.27	36.22 ± 0.14	36.20 ± 0.24
D-7	73.85 ± 6.19	62.72 ± 4.84	36.13 ± 0.21	35.92 ± 0.61
D-6	78.70 ± 6.27	94.40 ± 4.94	35.90 ± 0.35	36.05 ± 0.30
D-5	101.30 ± 5.30	130.75 ± 10.44	36.15 ± 0.19	36.21 ± 0.41
D-4	88.89 ± 6.03	128.54 ± 11.81	36.17 ± 0.26	36.17 ± 0.25
D-3	94.18 ± 6.26	152.59 ± 4.85	36.02 ± 0.39	36.01 ± 0.21
D-2	93.85 ± 4.65	141.41 ± 7.02	36.08 ± 0.25	36.18 ± 0.39
D-1	95.01 ± 7.07	145.69 ± 4.89	36.28 ± 0.23	36.01 ± 0.49
Diurnality score				
D-Pre	0.38 ± 0.03	0.38 ± 0.03		
D-7	0.38 ± 0.06	0.43 ± 0.07		
D-6	0.40 ± 0.04	0.67 ± 0.12		
D-5	0.36 ± 0.05	0.83 ± 0.06		
D-4	0.36 ± 0.04	0.72 ± 0.19		
D-3	0.32 ± 0.05	1.05 ± 0.06		
D-2	0.35 ± 0.06	0.89 ± 0.13		
D-1	0.31 ± 0.03	0.89 ± 0.12		

#### 3.2.2 Misaligned feeding disrupted pre-anaesthetic core body temperature rhythm

**Figure 3:**
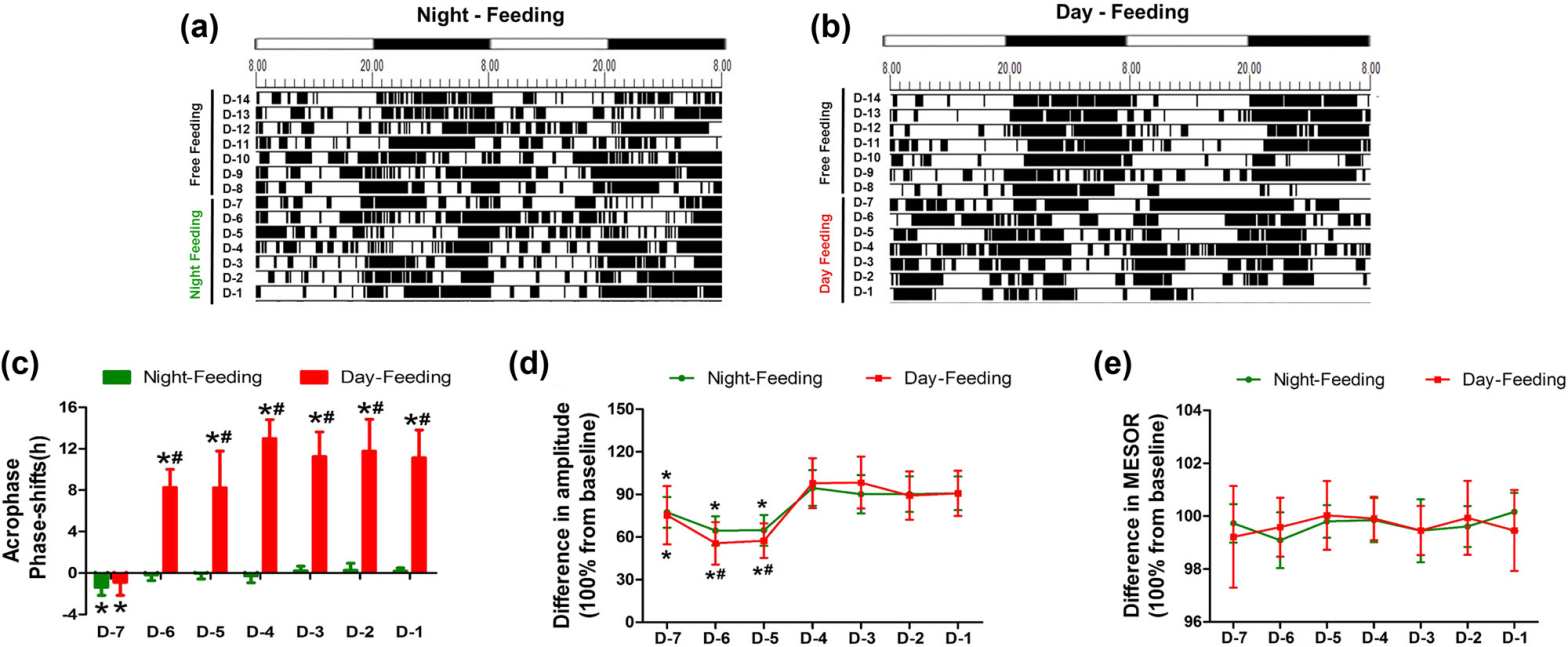
The effect of feeding schedule on temperature rhythm during 7 days. (a and b) Representative double-plotted actograms show the temperature patterns after feeding schedule in a 12 h:12 h light:dark cycle. The black sections in the actograms represent higher temperature than the average value in 48 h, while the white ones represent lower temperature, the abscissa 8.00 means ZT0; 20.00 means ZT12. (c) Phase shift of acrophase. (d) Amplitude. (e) MESOR. *P < 0.05 vs mean values in 7-day pre-feeding period; ^#^ *P* < 0.05 vs Night-Feeding group, *n* = 8.

The core body temperature patterns of the mice were analysed with Chronos-Fit and were displayed as double-plotted actograms (Figure 3a and b). The black sections of the actograms represent higher temperatures than the average value over 48 h, while the white sections represent lower temperatures. The rhythm of the core body temperatures had different trends of change between the Day-Feeding and the Night-Feeding mice. After several days of fluctuations, the Night-Feeding group adapted to the shift in food access gradually. The Day-Feeding group showed increased durations of higher temperatures in the daytime and decreased durations of higher temperatures at nighttime, suggesting that their nocturnality was disrupted. The temperature phase shift from baseline differed significantly between the Day-Feeding and the Night-Feeding groups. The Night-Feeding group showed phase delays on D-7 (20.69 ± 0.61, *P* < 0.001), which then reversed on D-6. The Day-Feeding group showed phase delays on D-7 (20.91 ± 0.81, *P* < 0.001), which then phase-inverted gradually over the subsequent days (D-6, 7.85 ± 1.27, *P* < 0.001; D-5, 9.33 ± 1.26, *P* < 0.001; D-4, 5.45 ± 1.62, *P* < 0.001; D-3, 8.75 ± 1.8, *P* < 0.001; D-2, 7.13 ± 1.37, *P* < 0.001; and D-1, 7.92 ± 1.39, *P* < 0.001) (Table 2). The Night-Feeding group exhibited a decreased amplitude on D-7–D-5 (D-7, 0.61 ± 0.08, *P* < 0.001; D-6, 0.51 ± 0.08, *P* < 0.001; and D-5, 0.51 ± 0.08, *P* < 0.001), an effect that reversed on D-4. The Day-Feeding group demonstrated a decreased amplitude of the temperature rhythm on D-7–D-5 (D-7, 0.58 ± 0.13, *P* = 0.007; D-6, 0.63 ± 0.13, *P* = 0.001; D-5, 0.61 ± 0.14, *P* = 0.002), which then reversed on D-4. For both the Night-Feeding and the Day-Feeding groups, the food schedule had no significant effect on the MESOR of body temperature rhythm.

### 3.3 RF altered post-anaesthetic daily rhythm levels

#### 3.3.1 RF altered the post-anaesthetic rest-activity rhythm

From the actograms, the Night-Feeding group and the Night-Feeding + Anes group showed transient rhythm fluctuations, while the Day-Feeding group gradually normalized their rhythm in the subsequent days; the Day-Feeding + Anes group showed persistently increased activity durations in the daytime and decreased activity durations at nighttime. After the RF protocol was omitted, the Night-Feeding group exhibited no rhythm parameter changes; the Day-Feeding group advanced to the acrophase on D1–D3, their amplitude decreased on D1 and D2, and MESOR decreased on D1–D4 and then recovered during the subsequent days.

**Figure 4:**
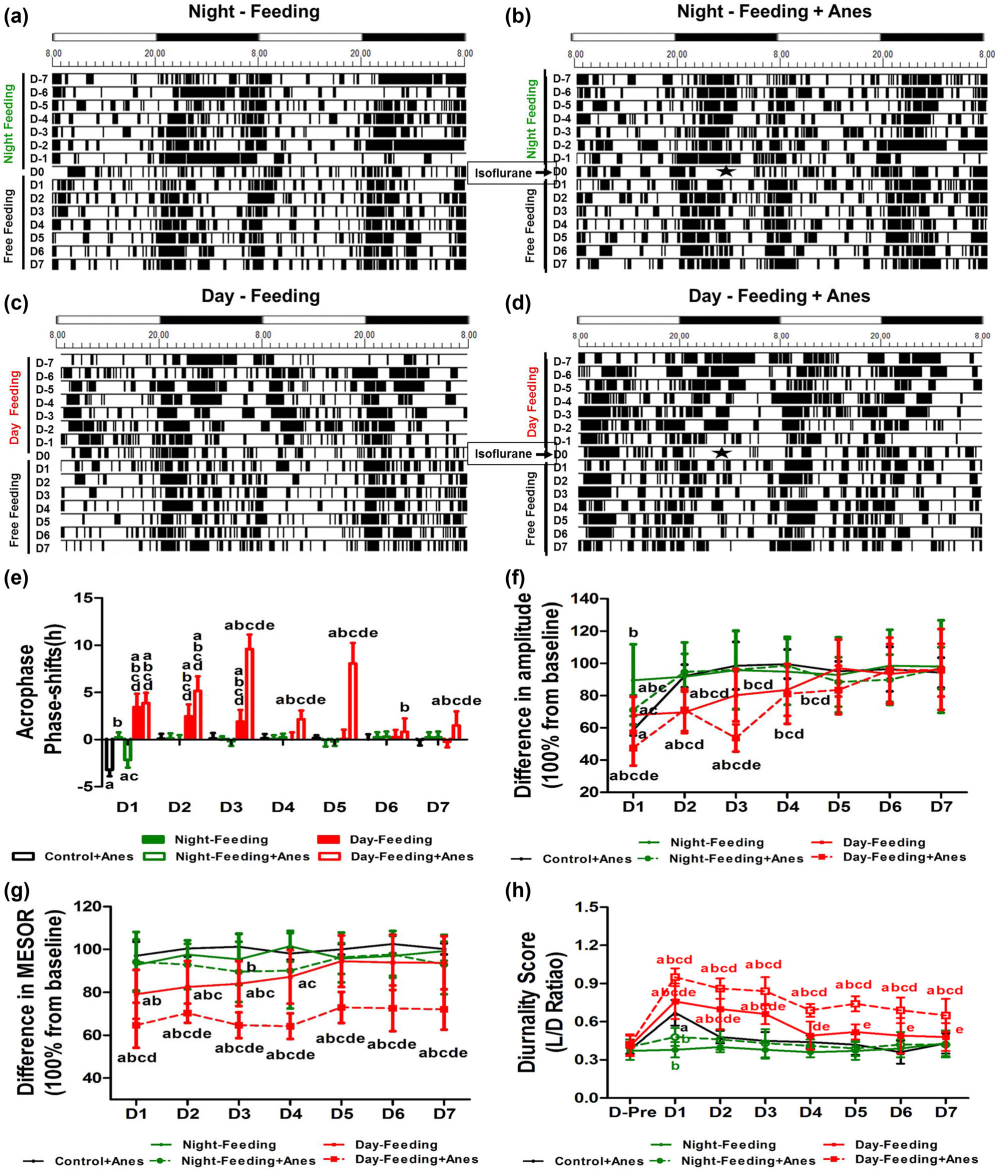
The effect of feeding schedule and isoflurane anaesthesia on rest-activity rhythm during 7 days (D1, D2, D3, D4, D5, D6, D7). (a–d) Representative double-plotted actograms show the activity patterns after feeding schedule and isoflurane anaesthesia in a 12 h:12 h light:dark cycle. The black sections in the actograms represent a greater amount of activity than the average value in 48 h, while the white ones represent less activity, the abscissa 8.00 means ZT0; 20.00 means ZT12. Pentagram represents anaesthesia administration from ZT14 to ZT19. (e) Phase shift of acrophase. (f) Amplitude. (g) MESOR. (h) Diurnality score. ^a^ *P* < 0.05 vs mean values in 7-day pre-administration period; ^b^ *P* < 0.05 vs Control + Anes group; ^c^ *P* < 0.05 vs Night-Feeding group; ^d^ *P* < 0.05 vs Night-Feeding + Anes group; ^e^ *P* < 0.05 vs Day-Feeding group, *n* = 8.

Multivariate repeated-measure analysis (between factors: food and anaesthesia); within factor: days on rest-activity rhythm acrophase showed significant effects of days (*F*_7,38_ = 12.73, *P* < 0.001), with significant interaction (days × food: *F*_14,78_ = 5.89, *P* < 0.001 and days × anaesthesia: *F*_7,38_ = 3.8, *P* = 0.03). After isoflurane anaesthesia, the Control + Anes group exhibited a delayed acrophase on D1 (21.58 ± 1.02, *P* < 0.001), a decreased amplitude on D1 (32.27 ± 4.18, *P* < 0.001), and an increased diurnality score on D1 (0.67 ± 0.1, *P* < 0.05), which then recovered on D2. The acrophase of the Night-Feeding + Anes group was also delayed on D1 compared to the baseline but restored approximately 1 h relative to that of the Anes group (20.39 ± 0.57, *P* < 0.001). The Day-Feeding + Anes group showed fluctuating phase advances from baseline on D1–D7 (D1, 14.36 ± 1.38, *P* < 0.001; D2, 13.07 ± 1.3, *P* < 0.001; D3, 8.62 ± 1.35, *P* < 0.001; D4, 16.08 ± 0.82, *P* < 0.001; D5, 10.15 ± 1.89, *P* < 0.001; D6, 17.40 ± 1.14, *P* < 0.01; and D7, 16.74 ± 1.55, *P* = 0.026), which was more difficult to reverse (Figure 4 and Table 3). Multivariate repeated-measure analysis (between factors: food and anaesthesia); within factor: days on rest-activity rhythm amplitude showed significant effects of days (*F*_7,38_ = 43.61, *P* < 0.001), food (*F*_16,76_ = 6.75, *P* < 0.001), and anaesthesia (*F*_8,37_ = 14.18, *P* < 0.001) with significant interaction (days × food: *F*_14,78_ = 4.20, *P* < 0.001 and days × anaesthesia: *F*_7,38_ = 10.66, *P* < 0.001). The amplitude of the Night-Feeding + Anes group decreased on D1 but recovered by approximately 11% compared with that of the Control + Anes group (38.5 ± 4.26, *P* = 0.003 vs. Baseline, *P* = 0.026 vs. Control + Anes) and reversed on D2. The amplitude of the Day-Feeding + Anes group decreased on D1–D3 (D1, 24.97 ± 3.7, *P* < 0.001; D2, 37.61 ± 2.66, *P* < 0.001; D3, 28.51 ± 2.86, *P* < 0.001), which then reversed on D4. Multivariate repeated-measure analysis (between factors: food and anaesthesia); within factor: days on rest-activity rhythm MESOR showed significant effects of days (*F*_7,38_ = 12.71, *P* < 0.001), food (*F*_16,76_ = 51.89, *P* < 0.001), and anaesthesia (*F*_8,37_ = 19.58, *P* < 0.001) with significant interaction (days × food: *F*_14,78_ = 3.28, *P* < 0.001). The MESOR of the Night-Feeding + Anes group did not change after isoflurane anaesthesia. The MESOR of the Day-Feeding + Anes group significantly decreased on D1–D7 (D-7, 62.12 ± 10.78, *P* < 0.001; D-6, 67.51 ± 5.02, *P* < 0.001; D-5, 61.95 ± 4.51, *P* < 0.001; D-4, 61.59 ± 5.06, *P* < 0.001; D-3, 69.91 ± 6.16, *P* < 0.001; D-2, 69.35 ± 7.79, *P* < 0.001; and D-1, 68.78 ± 5.67, *P* < 0.001). Multivariate repeated-measure analysis (between factors: food and anaesthesia); within factor: days on rest-activity rhythm diurnality score showed significant effects of days (*F*_7,38_ = 12.73, *P* < 0.001), food (*F*_16,76_ = 82.66, *P* < 0.001), and anaesthesia (*F*_8,37_ = 8.21, *P* < 0.001) with significant interaction (days × food: *F*_14,78_ = 5.89, *P* < 0.001 and days × anaesthesia: *F*_7,38_ = 3.8, *P* = 0.003). The diurnality score of the Day-Feeding + Anes group increased progressively on D1–D7.

**Table 3:** Results of post-anaesthetic locomotor activity cosinor analysis after RF

Locomotor activity					
	Control + Anes	Night-Feeding + Anes	Night-Feeding	Day-Feeding + Anes	Day-Feeding
Acrophase					
D-Pre	18.36 ± 0.41	18.36 ± 0.41	18.23 ± 0.31	18.18 ± 0.63	18.23 ± 0.44
D1	21.58 ± 1.02	18.13 ± 0.31	20.39 ± 0.57	14.74 ± 1.53	14.36 ± 1.38
D2	18.26 ± 0.42	18.23 ± 0.47	18.23 ± 0.45	15.71 ± 1.35	13.07 ± 1.30
D3	18.23 ± 0.44	18.35 ± 0.33	18.40 ± 0.70	16.27 ± 0.80	8.62 ± 1.35
D4	18.23 ± 0.31	18.31 ± 0.36	17.98 ± 0.27	18.19 ± 0.40	16.08 ± 0.82
D5	18.18 ± 0.63	18.38 ± 0.56	18.42 ± 0.24	18.14 ± 0.75	10.15 ± 1.89
D6	18.36 ± 0.29	18.13 ± 0.33	17.92 ± 0.57	17.93 ± 0.34	17.40 ± 1.14
D7	18.37 ± 0.44	18.13 ± 0.42	17.98 ± 0.46	18.41 ± 0.52	16.74 ± 1.55
Amplitude					
D-Pre	55.16 ± 7.52	57.66 ± 11.11	54.59 ± 5.72	52.49 ± 7.75	54.07 ± 8.41
D1	32.27 ± 4.18	49.62 ± 4.47	38.50 ± 4.26	34.96 ± 2.02	24.97 ± 3.70
D2	50.66 ± 6.38	51.3 ± 6.81	51.10 ± 1.54	35.82 ± 3.56	37.61 ± 2.66
D3	54.07 ± 8.41	52.99 ± 2.71	51.52 ± 9.92	41.34 ± 5.78	28.51 ± 2.86
D4	54.59 ± 5.72	52.99 ± 5.16	53.22 ± 6.84	43.26 ± 7.47	42.96 ± 6.83
D5	52.49 ± 7.75	51.48 ± 5.33	47.96 ± 7.89	49.95 ± 5.39	44.18 ± 4.08
D6	52.49 ± 4.54	55.13 ± 7.17	48.88 ± 9.27	47.80 ± 2.86	50.28 ± 4.52
D7	51.57 ± 5.38	54.2 ± 8.95	52.66 ± 6.74	49.10 ± 9.10	51.18 ± 7.82
MESOR					
D-Pre	95.29 ± 7.32	96.37 ± 4.97	94.77 ± 6.70	95.40 ± 8.53	96.19 ± 6.33
D1	92.14 ± 5.54	88.93 ± 8	88.95 ± 12.66	75.05 ± 10.11	62.12 ± 10.78
D2	95.76 ± 9.27	93.85 ± 2.49	87.37 ± 6.55	78.12 ± 9.23	67.51 ± 5.02
D3	96.19 ± 6.33	91.45 ± 7.91	84.14 ± 9.22	79.64 ± 8.01	61.95 ± 4.51
D4	93.52 ± 8.70	97.68 ± 7.25	84.55 ± 13.26	82.54 ± 9.38	61.59 ± 5.06
D5	95.40 ± 8.53	92.03 ± 4.93	90.82 ± 9.52	89.43 ± 7.63	69.91 ± 6.16
D6	97.44 ± 5.20	93.2 ± 7.43	91.95 ± 6.23	88.80 ± 7.80	69.35 ± 7.79
D7	95.4 ± 6.57	95.42 ± 3.52	87.33 ± 8.26	88.76 ± 9.06	68.78 ± 5.67
Diurnality score					
D-Pre	0.38 ± 0.03	0.40 ± 0.06	0.37 ± 0.07	0.42 ± 0.08	0.41 ± 0.08
D1	0.67 ± 0.10	0.48 ± 0.07	0.38 ± 0.06	0.95 ± 0.07	0.76 ± 0.14
D2	0.48 ± 0.05	0.46 ± 0.08	0.40 ± 0.04	0.86 ± 0.08	0.70 ± 0.16
D3	0.45 ± 0.07	0.43 ± 0.11	0.38 ± 0.07	0.84 ± 0.11	0.66 ± 0.08
D4	0.44 ± 0.05	0.41 ± 0.05	0.36 ± 0.04	0.69 ± 0.05	0.49 ± 0.11
D5	0.42 ± 0.08	0.39 ± 0.06	0.37 ± 0.07	0.74 ± 0.06	0.52 ± 0.06
D6	0.46 ± 0.09	0.42 ± 0.10	0.39 ± 0.07	0.69 ± 0.1	0.49 ± 0.14
D7	0.43 ± 0.08	0.42 ± 0.10	0.43 ± 0.07	0.65 ± 0.13	0.48 ± 0.11

#### 3.3.2 RF altered post-anaesthetic core body temperature rhythm

From the actograms, we found that the core body temperatures of the different groups had similar trends of change in terms of locomotor activity. The acrophase of the Day-Feeding group advanced on D1–D3, the amplitude decreased on D1–D5, which then recovered over the subsequent days. The MESOR of the Day-Feeding showed no change.

**Figure 5:**
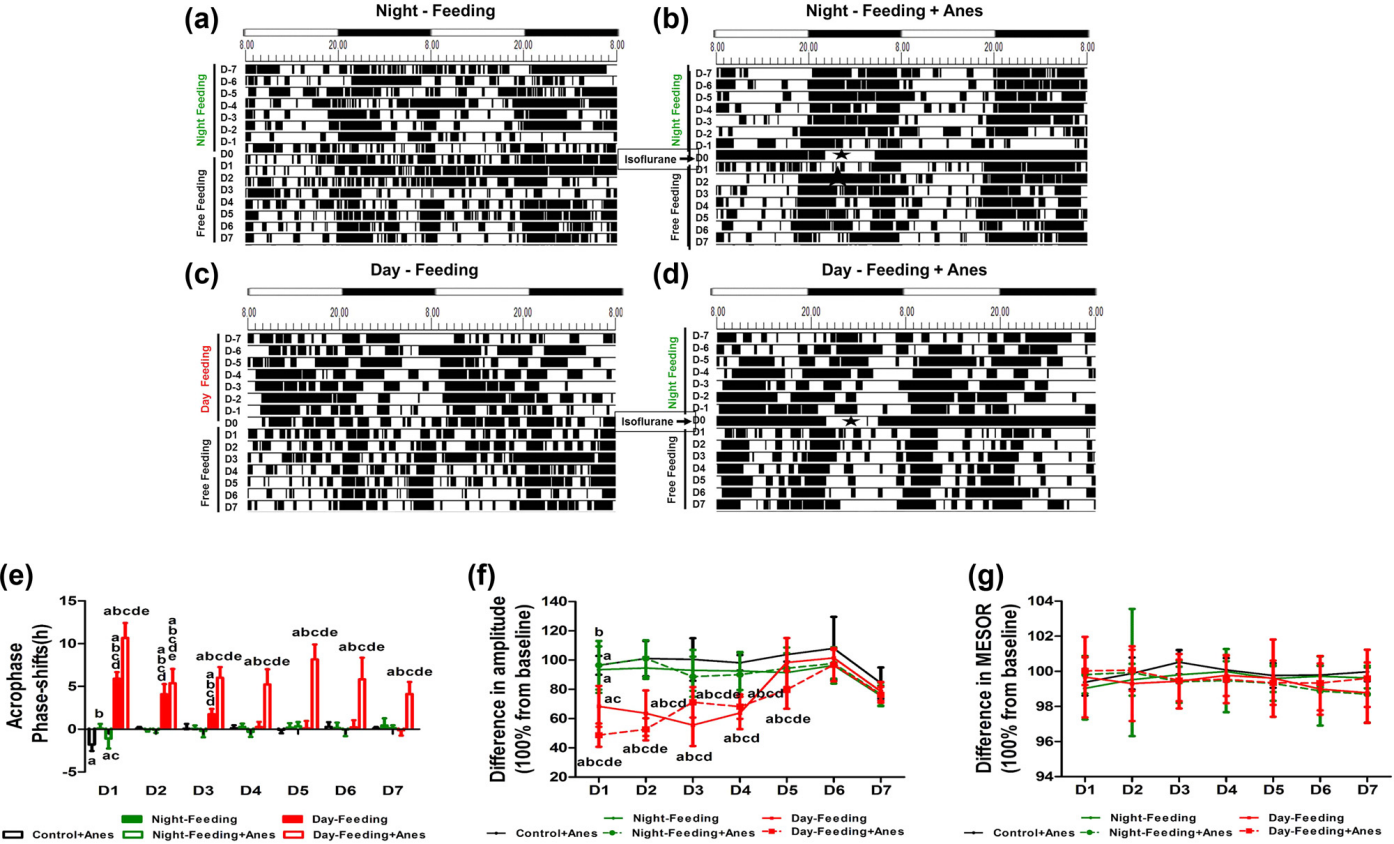
The effect of feeding schedule and isoflurane anaesthesia on temperature rhythm during 7 days (D1, D2, D3, D4, D5, D6, D7). (a–d) Representative double-plotted actograms show the temperature patterns after feeding schedule and isoflurane anaesthesia in a 12 h:12 h light:dark cycle. The black sections in the actograms represent higher temperature than the average value in 48 h, while the white ones represent lower temperature, the abscissa 8.00 means ZT0; 20.00 means ZT12. Pentagram represents anaesthesia administration from ZT14 to ZT19. (e) Phase shift of acrophase. (f) Amplitude. (g) MESOR. ^a^ *P* < 0.05 vs Mean values in 7-day pre-administration period; ^b^ *P* < 0.05 vs Control + Anes group; ^c^ *P* < 0.05 vs Night-Feeding group; ^d^ *P* < 0.05 vs Night-Feeding + Anes group; ^e^ *P* < 0.05 vs Day-Feeding group, *n* = 8.

Multivariate repeated-measure analysis (between factors: food and anaesthesia); within factor: days on temperature acrophase showed significant effects of days (*F*_7,38_ = 31.17, *P* < 0.001), food (*F*_16,76_ = 48.73, *P* < 0.001), and anaesthesia (*F*_8,37_ = 13.17, *P* = 0.001) with significant interaction (days × food: *F*_14,78_ = 6.37, *P* < 0.001 and days × anaesthesia: *F*_7,38_= 15.65, *P* < 0.001). After isoflurane anaesthesia, the Control + Anes group exhibited a delayed acrophase on D1 (20.21 ± 0.74, *P* < 0.001), a decreased amplitude on D1 (0.50 ± 0.08, *P* < 0.001), which then recovered on D2. The Night-Feeding + Anes group acrophase was delayed on D1 (20.46 ± 0.91, *P* < 0.001), compared with the baseline. The Day-Feeding + Anes group showed persistent phase advances from baseline on D1–D7 (D1, 7.47 ± 1.69, *P* < 0.001; D2, 12.78 ± 1.42, *P* < 0.001; D3, 12.15 ± 1.15, *P* < 0.001; D4, 12.92 ± 1.58, *P* < 0.001; D5, 9.99 ± 1.69, *P* < 0.001; D6, 12.33 ± 2.32, *P* < 0.001; and D7, 14.05 ± 1.26, *P*< 0.001), an effect that was difficult to reverse (Figure 5 and Table 4). Multivariate repeated-measure analysis (between factors: food and anaesthesia); within factor: days on temperature amplitude showed significant effects of days (*F*_7,38_ = 40.21, *P* < 0.001), food (*F*_16,76_ = 143.18, *P* < 0.001) with significant interaction (days × food: *F*_14,78_ = 5.32, *P* < 0.001 and days × anaesthesia: *F*_7,38_ = 16.90, *P* < 0.001). The Night-Feeding + Anes group’s amplitude decreased on D1 (0.46 ± 0.03, *P* < 0.001), an effect that was reversed on D2. The Day-Feeding + Anes group exhibited a decreased amplitude in the temperature rhythm on D1–D5 (D1, 0.36 ± 0.04, *P* < 0.001; D2, 0.38 ± 0.04, *P* < 0.001; D3, 0.41 ± 0.04, *P* < 0.001; D4, 0.56 ± 0.06, *P* = 0.001; and D5, 0.54 ± 0.04, *P* < 0.001), an effect that was reversed on D6. For both the Night-Feeding + Anes and the Day-Feeding + Anes groups, the food schedule had no significant effect on the MESOR of the body temperature rhythm on D1–D7.

**Table 4:** Results of post-anaesthetic body core temperature cosinor analysis after RF

Temperature					
	Control + Anes	Night-Feeding + Anes	Night-Feeding	Day-Feeding + Anes	Day-Feeding
Acrophase					
D-Pre	18.42 ± 0.25	18.42 ± 0.25	18.31 ± 0.24	18.48 ± 0.62	18.15 ± 0.41
D1	20.21 ± 0.74	18.32 ± 0.42	20.46 ± 0.91	12.57 ± 0.52	7.47 ± 1.69
D2	18.33 ± 0.39	18.45 ± 0.34	18.35 ± 0.26	14.39 ± 1.09	12.78 ± 1.42
D3	18.40 ± 0.56	18.39 ± 0.39	18.50 ± 0.72	16.73 ± 0.19	12.15 ± 1.15
D4	18.31 ± 0.24	18.15 ± 0.30	18.61 ± 0.54	18.22 ± 0.45	12.92 ± 1.58
D5	18.48 ± 0.62	18.22 ± 0.40	17.96 ± 0.56	18.36 ± 0.46	9.99 ± 1.69
D6	18.15 ± 0.42	18.27 ± 0.53	18.33 ± 0.75	18.25 ± 0.54	12.33 ± 2.32
D7	18.33 ± 0.17	17.97 ± 0.73	18.14 ± 0.27	18.61 ± 0.42	14.05 ± 1.26
Amplitude					
D-Pre	0.79 ± 0.08	0.79 ± 0.08	0.79 ± 0.08	0.78 ± 0.09	0.80 ± 0.08
D1	0.50 ± 0.08	0.73 ± 0.08	0.46 ± 0.03	0.50 ± 0.05	0.36 ± 0.04
D2	0.76 ± 0.08	0.73 ± 0.09	0.75 ± 0.07	0.52 ± 0.06	0.38 ± 0.04
D3	0.80 ± 0.08	0.75 ± 0.05	0.79 ± 0.05	0.49 ± 0.08	0.41 ± 0.04
D4	0.79 ± 0.08	0.73 ± 0.08	0.69 ± 0.06	0.42 ± 0.08	0.56 ± 0.06
D5	0.78 ± 0.09	0.73 ± 0.06	0.70 ± 0.08	0.49 ± 0.06	0.54 ± 0.04
D6	0.82 ± 0.10	0.71 ± 0.07	0.73 ± 0.07	0.75 ± 0.06	0.63 ± 0.07
D7	0.84 ± 0.11	0.76 ± 0.07	0.77 ± 0.08	0.79 ± 0.06	0.76 ± 0.05
MESOR					
D-Pre	36.21 ± 0.14	36.21 ± 0.14	36.24 ± 0.14	36.21 ± 0.33	36.20 ± 0.14
D1	35.98 ± 0.17	35.86 ± 0.68	36.17 ± 0.38	36.08 ± 0.62	36.20 ± 0.26
D2	36.17 ± 0.28	36.03 ± 0.32	36.21 ± 1.24	35.95 ± 0.63	36.22 ± 0.35
D3	36.40 ± 0.14	36.14 ± 0.15	36.02 ± 0.41	36.00 ± 0.38	36.01 ± 0.37
D4	36.24 ± 0.14	36.20 ± 0.16	36.04 ± 0.61	36.12 ± 0.19	36.03 ± 0.44
D5	36.12 ± 0.19	36.06 ± 0.27	35.99 ± 0.29	36.06 ± 0.55	35.96 ± 0.40
D6	36.13 ± 0.13	36.11 ± 0.19	35.83 ± 0.63	35.84 ± 0.41	35.95 ± 0.50
D7	36.20 ± 0.08	36.07 ± 0.16	35.76 ± 0.49	35.77 ± 0.47	36.05 ± 0.54

### 3.4 Misaligned feeding prolonged post-anaesthetic hippocampus-independent learning and memory deficits

**Figure 6:**
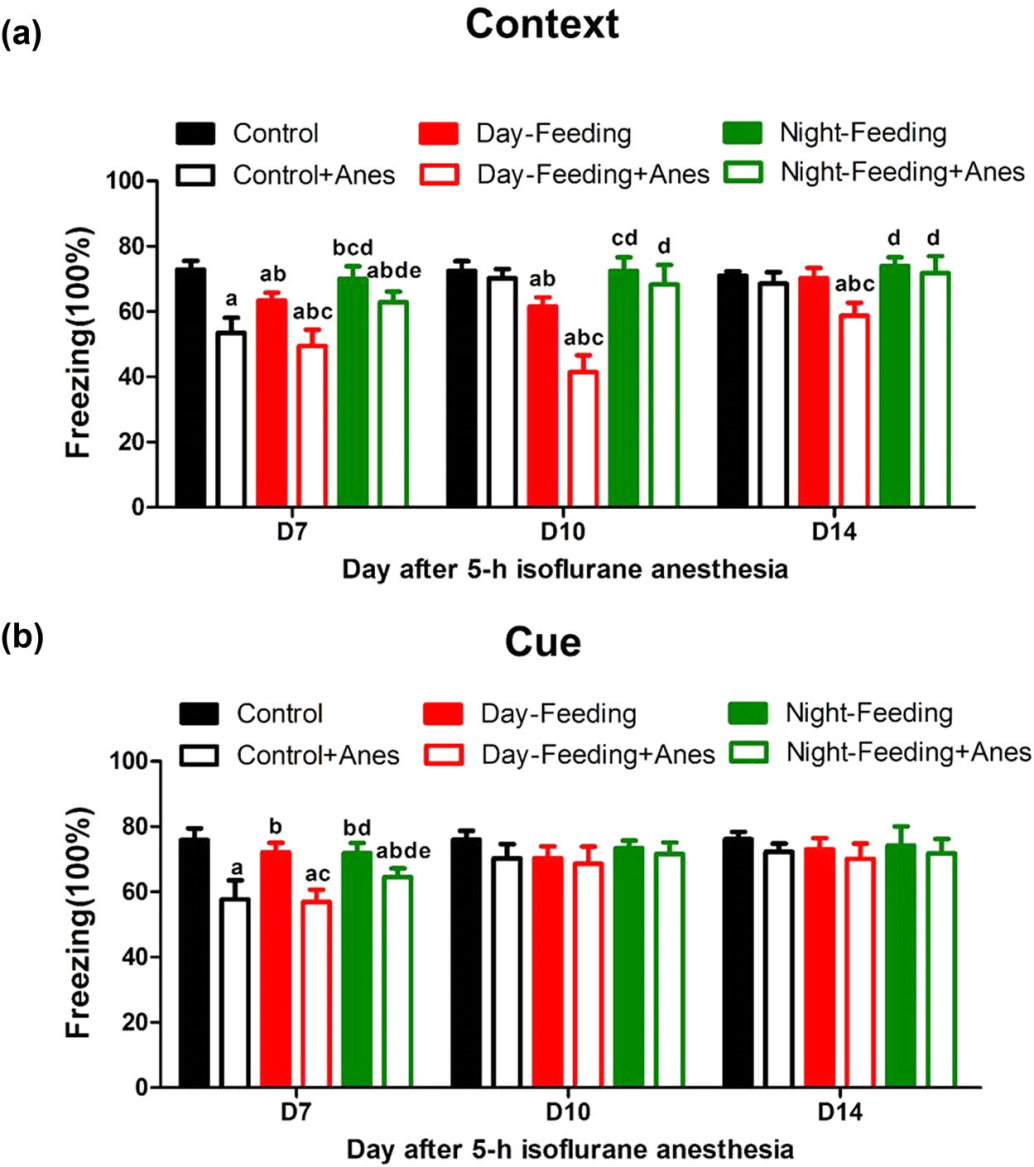
The effect of 7-day feeding schedule on post-anaesthetic fear memory at D7, D10, D14. (a) Contextual fear conditioning. Daytime feeding and isoflurane induced persistent contextual fear memory decline on D7, D10, D14. (b) Cued-fear conditioning. Isoflurane induced cued-fear memory decline on D7. ^a^ *P* < 0.05 vs Control; ^b^ *P* < 0.05 vs Control + Anes group; ^c^ *P* < 0.05 vs Day-Feeding group; ^d^ *P* < 0.05 vs Day-Feeding + Anes group; ^e^ *P* < 0.05 vs Night-Feeding + Anes group; *n* = 8.

Compared with the Control group, the Control + Anes group showed a contextual memory decrease on D7 (53.43 ± 4.73%, *P* < 0.001) and then recovered on D10 (70.18 ± 2.82%, *P* > 0.05). The Day-Feeding group exhibited a reduction in contextual fear-conditioned behaviour, which lasted until D10 (D7, 63.34 ± 2.47%, *P* < 0.001; and D10, 61.58 ± 2.81%, *P* < 0.001) and recovered on D14 (70.22 ± 3.17%, *P* > 0.05), indicating that the circadian misalignment damaged hippocampus-dependent memory. There was no difference between the Night-Feeding group and the Control group. Compared to the Control + Anes group, the Night-Feeding + Anes group showed an increase in contextual fear memory on D7 (62.86 ± 3.24%, *P* < 0.001), though this increase was still lower than that of the Control group (*P* < 0.001). The Day-Feeding group did not exhibit aggravated memory deficits on D14, compared with the Control group (*P* = 0.647). However, compared with the Control group, the Day-Feeding + Anes group showed a prolonged memory deficit, lasting even 2 weeks post-anaesthesia (D7, 49.49 ± 4.95%, *P* < 0.001; D10, 46.16 ± 5.46%, *P* < 0.001; and D14, 58.72 ± 3.97%, *P* < 0.001), with the Control + Anes group recovering on D10 and the Day-Feeding group recovering on D14. The Day-Feeding + Anes group showed reduced contextual memory compared to the Day-Feeding group on D7 (*P* < 0.001), D10 (*P* < 0.001), and D14 (*P* < 0.001); the Night-Feeding + Anes group also showed reduced contextual memory compared to the Night-Feeding group on D7 (*P* = 0.007), which suggested that isoflurane anaesthesia aggravated the misaligned feeding-induced hippocampus-dependent memory dysfunction. Amygdala-dependent cued-fear conditioning was not altered by the alignment/misalignment of feeding (Figure 6b). Isoflurane induced cued memory deficits on D7 (*P* < 0.001).

## 4 Discussion

In this study, we sought to determine if temporally RF schedules in mice could impact post-anaesthetic cognition through daily rhythm modulation. We proved that a 1-week short period of misaligned food before anaesthesia was sufficient to lead to sustained post-anaesthetic daily rhythm disruption; meanwhile, aligned feeding partially restored post-anaesthetic daily rhythm parameters. One-week aligned feeding attenuated and even reversed post-anaesthetic memory deficits, while 1 week misaligned feeding led to substantial post-anaesthetic hippocampus-dependent contextual fear conditioning impairment, even when the mice were switched back to *ad libitum* feeding.

In our experiments, the mice did not lose or gain weight. Therefore, the present study emphasized how short-term disrupted feeding schedules induced daily rhythm disturbance, instead of metabolic effects. Animal studies [14,15] have shown that food intake can serve as a major time cue for peripheral tissue clocks, such as the liver, but not for the SCN. Conflicting zeitgeber timings can lead to internal desynchrony, a state in which different clocks throughout the body are out of sync [16]. In this experiment, we quantitated several parameters that characterize daily rhythm: the acrophase, amplitude, and MESOR, along with diurnality score. Alterations in these measures indicate impairments in the regulation of the circadian system [17]. The daily rhythms of locomotor activity and body temperature have been simultaneously monitored in many studies conducted on laboratory animals [18,19]. Our results showed a daily nocturnal rhythm of body temperature and locomotor activity: longer duration of higher values during the night and shorter duration of higher values during the day; the rhythm of body temperature proceeding closely to the rhythm of locomotor activity, except in regard to the MESOR parameter. The MESOR of the different rhythms could not be compared because they refer to distinct physical quantities. The robustness of rhythm should be taken into consideration.

Loh reported that 2 weeks of ZT3 to ZT9 food access alters diurnal rhythms of activity and sleep [20]. In our experiments, before anaesthesia, with 7 days of feeding during the subjective rest time, the acrophase of locomotor activity converted 18.21 to 7.19, and the diurnality score converted 0.38–0.89, which suggested that we successfully modify the basic daily rhythm. Others have suggested that feeding-entrained rhythms persist for several days, if feedings are omitted [21]. Consistently, we confirmed that after 1 week of restricted food, the locomotor activity and temperature rhythm parameters mainly returned to baseline (Figures 4 and 5).

Post-operative patients usually experience various sleep disturbances, including sleep-wake cycle disturbances [22]. There have been an increasing number of studies that have demonstrated that anaesthetics can disrupt the daily rhythm [23,24,25]. We also proved in this experiment that long-term isoflurane anaesthesia caused mice with *ad libitum* feeding an approximately 3 h acrophase delay and 40% amplitude decrease, and 76% diurnality score increase on D1 and returned back to baseline on D2. Nowadays, many people live in an environment where their circadian system is challenged by inappropriate work-times associated with dietary intake. In this study, we manipulated feeding time shifts to explore their effects on post-anaesthetic daily rhythm. Daytime feeding for 1 week caused aggravated post-anaesthetic daily rhythm parameter disruption, which lasted for approximately 5–7 days; nighttime feeding partially recovered post-anaesthetic acrophase shift, the amplitude decrease, and diurnality score increase. Therefore, we demonstrated that 7 days of misaligned feeding was sufficient to induce basic circadian dysrhythmia, which then aggravated and prolonged the post-anaesthetic circadian rhythmicity disruption. Meanwhile, aligned feeding partially enhanced post-anaesthetic daily rhythm.

Circadian rhythms modulate many physiological behaviours, including memory processes [26]. Long-term exposure to internal desynchrony is thought to contribute to the adverse effects of circadian misalignment, including the increased risk for cognitive dysfunction [27,28]. Some pieces of clinical evidence have suggested that mealtime interventions improve behavioural symptoms in elderly people with dementia [29,30]. In our previous work, we also found that melatonin pre-treatment prevented isoflurane-induced cognitive dysfunction by modulating sleep-wake rhythms in mice. Isoflurane anaesthesia induced contextual and cue memory dysfunction on D7 and then recovered on D10 in mice. Therefore, we tried to explore whether a 1 week aligned feeding schedule was sufficient to enhance post-anaesthetic daily rhythm, which could ameliorate post-anaesthetic memory deficits. Then, we proved that feeding at activity time for 1 week improved post-anaesthetic memory on D7. We did not see aggravated post-anaesthetic memory deficits after daytime feeding. A reason for this may have been a “floor effect”, as misaligned feeding could not aggravate the sharp memory decrease induced by isoflurane anaesthesia. Another explanation may be that FCS was not sufficient for the detection of precise differences between the two groups. Automatically, we lengthened the observation time and sought to determine whether pre-anaesthetic daily rhythm disturbance could prolong post-anaesthetic cognitive dysfunction. We proved that 1 week of feeding during rest time prolonged post-anaesthetic contextual memory deficits, which lasted 2 weeks, with feeding being omitted after anaesthesia, whereas the memory of the wild-type recovered 10 days after isoflurane anaesthesia. Compared with the contextual memory test, RF did not change amygdala-dependent cued-fear conditioning. This implied that some learned behaviours are more vulnerable to the impact of misaligned feeding. Potential mechanisms should be considered in the future study. A possible mechanism may be 5-HT_7_ receptor, a subtype of the 5-HT receptor family, and is highly expressed in the hippocampus and SCN, where it is strongly involved in cognition and diurnal–nocturnal rhythm of animal’s activity.

Our study demonstrated that an incorrect time of food intake and the subsequent circadian disruption might aggravate and prolong post-anaesthetic cognitive dysfunction. Meanwhile, aligned feeding induced robust rhythmicity and could improve post-anaesthetic memory deficits. Meal timing can be under individual control and thus represents a potentially complementary approach for future postoperative cognitive dysfunction prevention strategies.
